# Perceptions of Healthcare-Associated Infection and Antibiotic Resistance among Physicians Treating Syrian Patients with War-Related Injuries

**DOI:** 10.3390/ijerph15122709

**Published:** 2018-12-01

**Authors:** Andreas Älgå, Karin Karlow Herzog, Murad Alrawashdeh, Sidney Wong, Hamidreza Khankeh, Cecilia Stålsby Lundborg

**Affiliations:** 1 Department of Clinical Science and Education, Södersjukhuset, Karolinska Institutet, 11883 Stockholm, Sweden; hamid.khankeh@ki.se; 2 Department of Public Health Sciences, Karolinska Institutet, 17177 Stockholm, Sweden; karin.herzog@gotland.se (K.K.H.); cecilia.stalsby.lundborg@ki.se (C.S.L.); 3 Médecins Sans Frontières, Ar Ramtha 21410, Jordan; muradalrawashdeh@yahoo.com; 4 Médecins Sans Frontières, Operational Centre Amsterdam, 1018 DD Amsterdam, The Netherlands; sidney.wong@amsterdam.msf.org; 5 Health in Emergency and Disaster Research Centre, University of Social Welfare and Rehabilitation Sciences, Tehran 1985713834, Iran

**Keywords:** healthcare-associated infections, antibiotic resistance, resource-limited setting, war injuries, qualitative research

## Abstract

Healthcare-associated infections (HAIs) constitute a major contributor to morbidity and mortality worldwide, with a greater burden on low- and middle-income countries. War-related injuries generally lead to large tissue defects, with a high risk of infection. The aim of this study was to explore how physicians in a middle-income country in an emergency setting perceive HAI and antibiotic resistance (ABR). Ten physicians at a Jordanian hospital supported by Médecins Sans Frontières were interviewed face-to-face. The recorded interviews were transcribed verbatim and analyzed by qualitative content analysis with an inductive and deductive approach. The participants acknowledged risk factors of HAI and ABR development, such as patient behavior, high numbers of injured patients, limited space, and non-compliance with hygiene protocols, but did not express a sense of urgency or any course of action. Overuse and misuse of antibiotics were reported as main contributors to ABR development, but participants expressed no direct interrelationship between ABR and HAI. We conclude that due to high patient load and limited resources, physicians do not see HAI as a problem they can prioritize. The knowledge gained by this study could provide insights for the allocation of resources and development of hygiene and wound treatment protocols in resource-limited settings.

## 1. Introduction

Healthcare-associated infections (HAIs) constitute a major threat to patient safety worldwide by prolonging hospital stay, elevating rates of morbidity and mortality, and greatly increasing the financial burden [1]. Hundreds of millions of patients are affected by HAIs annually, with higher rates in low- and middle-income countries (LMICs) [2,3]. In these countries, patients and healthcare workers are vulnerable to HAIs and its consequences due to understaffing, overcrowding, and shortage of basic equipment such as gloves [4,5]. In addition, the situation is often aggravated by unsatisfactory hand hygiene [6,7,8,9,10,11,12].

Antibiotic resistance (ABR) is a growing problem worldwide and is considered by the World Health Organization (WHO) to “threaten the very core of modern medicine” [13]. The increasing spread of ABR is a particular problem in LMICs, where the burden of infections is high, antibiotics are often being inappropriately prescribed [14,15], and the supply of newer types of antibiotics is limited due to cost [16,17]. Antibiotic-resistant organisms causing HAI has been shown to be a major concern in LMICs [18].

Patients with traumatic injuries have an increased risk of developing infection [19] due to tissue damage and impaired host defense mechanisms [20]. In LMICs, the management of these injuries is complicated by the limited resources available. War-related injuries are associated with large tissue defects and a high risk of infection [21]. Data is scarce on how physicians treating patients with war-related injuries in LMICs view HAI and ABR. As part of a project to assess the challenges hospital-based physicians encounter in a middle-income country emergency setting [22], the aim of this study was to explore physicians’ experiences and perceptions on HAI and ABR.

## 2. Materials and Methods

Due to the explorative nature of this study, a qualitative study design using interviews and subsequent content analysis was selected. By letting participants freely present their views and elaborate on a topic, this design has shown to be effective in generating knowledge not possible to obtain by quantitative methods [23].

### 2.1. Study Setting

Jordan is an upper-middle-income country, [24] currently hosting over 655,000 Syrian refugees [25]. The Jordan Ministry of Health hospital in Ar Ramtha is located five kilometers from the Syrian border. At this hospital Médecins Sans Frontières/Doctors Without Borders (MSF) runs an emergency trauma project, providing care for civilians injured in the Syrian armed conflict. On average, 60 hospital beds are used, plus one bed at the ICU and two operating rooms. Nurses and non-specialized physicians working at the emergency department and the wards are from Jordan. The general and orthopedic surgeons and anesthesiologists are approximately 75% Jordanian and 25% MSF expatriates from all over the world. Among acutely injured civilians with wound infection, multidrug-resistant organisms have been found in 73% of wound cultures [26]. This is higher than in previous reports of Syrian civilians with war-related injuries treated in Jordan and Israel [27,28].

### 2.2. Participants

Practicing physicians at the MSF project in Ar Ramtha hospital were interviewed. Selection was made using purposeful sampling, to form a heterogeneous group in terms of age, sex, medical specialty, and working experience. Table 1 summarizes characteristics of the participants.

### 2.3. Data Collection

Semi-structured, individual, face-to-face interviews were conducted during 2015 in Ar Ramtha hospital, using an interview guide in English, used also for a previous study [22] (Appendix A). All interviews were conducted by the first author (A.Ä.), a general surgery resident that has worked primarily in Sweden, but also has MSF field experience. The participants were enrolled until the research group concluded that saturation was reached. In total, ten interviews were conducted, with a mean duration of 32 min.

### 2.4. Data Analysis

The recorded interviews were transcribed verbatim and analyzed by content analysis. The texts were read and re-read to correct misinterpretations and to grasp the over-all content. Member-check was utilized when clarification was needed. Manifest and latent content analysis was performed with an inductive and deductive approach, as described by Graneheim and Lundman [29]. For the deductive analysis, the views put forward by the participants were continuously compared with MSF protocols for wound care and hygiene. Meaning units were identified, condensed, and labelled with a code. Codes were compared and grouped, whereupon sub-categories and categories were developed, both by inductive and deductive approach using manifest content analysis. The main theme was derived through latent content analysis. Analysis was conducted using tables in Microsoft Word 2011 (Microsoft, Redmond, WA, USA).

### 2.5. Ethics

This study was performed in accordance with the Declaration of Helsinki, and the protocol was approved by the Ethics Review Committee of the Jordan Ministry of Health (MOH REC 150037) as well as by the MSF ethics review board (ID 1520). Written informed consent was obtained from all participants and information about the study was given at the start of each interview. The participants were informed that they could withdraw from the study at any time.

## 3. Results

One main theme emerged from the analysis: “Interrelationship between behavior, healthcare-associated infections, and the development of antibiotic resistance”. The theme presents factors related to the development of HAI and ABR, as well as their consequences. The theme emerged from two categories: (i) Healthcare-associated infections, and (ii) antibiotic resistance (Table 2). The findings are presented under each category/subcategory. Quotations are followed by participant ID number. Explanations by the authors are presented in squared brackets.

### 3.1. Healthcare-Associated Infections

This category was built from two subcategories: (i) Behaviors and conditions influencing the spread of infection, and (ii) lack of consensus on the problem. All participants presented behavioral factors they believed could affect the spread of infection. Staff behavior was recognized as a potential risk factor of spreading pathogens causing HAI, e.g., not following hand hygiene routines when performing dressing changes. On the other hand, staff behavior was also described as a way of limiting the spread of pathogens causing HAI. Examples of such behavior included increasing the distance between beds or implementing measures to improve hand hygiene. Patient and caregiver behavior, however, was generally perceived as increasing the spread of pathogens causing HAI, for example, by sharing everything in the wards and common spaces, even patient beds.

*“The dressing and the […] on the same bed you will expose the whole area around you with the same organism. When you remove the, when you remove the gauze you might just spread the colonization from the wound to the area around you. To the bed side, to the sheet, to the sheet of the bed, to the cupboard of the patient, without even touching the wound or touching them by your hand itself.”*

(2)

Participants expressed limited resources as a main reason for not adhering to protocols. Main concerns were a lack of supplies, such as bed sheets, correct types of antibiotics, and limited space for high numbers of patients, generating over-crowded wards with close proximity of beds. Participants perceived this lack of space as being associated with increased risk of spreading bacteria and a possible contributor to HAI.

Participants differed in their views on the significance of HAI. Some described it as a significant problem while others did not. Hand hygiene was mentioned both as a factor that does and does not affect the spread of HAI. One participant emphasized that the hand hygiene routines should not affect the level of HAI because most of the dressing changes were done in the operating theatre. Distinguishing between HAI and infections caused by contamination at the time of injury was also brought up as a challenge when assessing the level of HAI.

*“You know, honestly you cannot judge it because all war wounds are initially contaminated. Then if, after five days you have a contamination then, or infection, then you can blame it to the nosocomial or to the origin of the initial phase of the wound. And this will be a difficult to judge, I think. At least from my point of view.”*

(8)

### 3.2. Antibiotic Resistance

This category was constructed from two subcategories: (i) Causes of antibiotic resistance, and (ii) challenges related to antibiotic resistance. According to the participants, antibiotics can be bought over the counter without a prescription, both in Syria and in Jordan. This is described for both narrow and broad-spectrum antibiotics.

*“In Jordan, we have a big issue of the antibiotic and, I think Syria have the same problem. It’s something culturally based for everyone has any [health] issue. For example, [if] he had a normal sore throat he will just go to the pharmacy and buy the amoxicillin by himself, without a prescription. He will take three to four times and that’s it.”*

(2)

The participants describe behavior related to how antibiotics are used. Both the behavior of doctors and people in the community were considered as possible contributors to ABR. According to the participants, the duration of treatment could affect the development of ABR, both by prescribing long treatments but also if a treatment is interrupted. Prescribing antibiotics without an indication was also recognized as a possible cause. Over-use and over-the-counter purchase of antibiotics as well as discontinuing a course of antibiotics were presented as patient behavior leading to the development of ABR.

*“The misuse of antibiotics from the beginning. Or even for cases where it was not necessary and then changing antibiotics all the time. This might be a reason as well, that they are not given long enough, really to fight the infection.”*

(4)

According to the participants, the main challenges linked to ABR are the high cost of the antibiotics required for treatment, and the lack of space for isolation of patients with infections caused by antibiotic-resistant bacteria. The antibiotics needed to treat antibiotic-resistant bacteria were described both as more expensive but also more complicated to acquire. Isolation was primarily seen as problematic due to the already crowded wards.

*“So, because of long period of time and the type of antibiotics we, we use, yeah. They are expensive.”*

(10)

*“And this is a, of course cost effective and also, you know for example in our hospital we have maximum 40 patients. Sometimes we face three to four cases in need for isolation and this is another burden on hospital regarding isolation, apart from the cost of antibiotic; isolation.”*

(3)

## 4. Discussion

We found that physicians in a setting with high numbers of severely injured patients and limited resources do not prioritize issues related to HAI. A surprising finding was the lack of consensus among the participants on the seriousness of HAI. Some described it as a major problem, while others did not think it to be significant. These different views could in part be explained by the participants’ heterogeneity in terms of origin, training, and experiences, possibly leading to different expectations and interpretations of the level of HAI. One participant pointed out the difficulty of differentiating between infection and bacterial contamination at the time of injury, and that this difficulty leads to uncertainties when trying to assess the levels of HAI. This concern is recognized in previous research [30,31].

Contributors to HAI development brought forward by the participants included limited space, patient behavior, and insufficient adherence to protocols for wound care, antibiotic treatment, and hygiene. Participants acknowledged that there could be a link between failure to adhere to hygiene protocols and the development of HAI but expressed no further thoughts on the matter. We sensed that there were no strong standpoints on how to manage these issues. This void of discussion on a course of action might be a demonstration of the trade-off having to be made when working in a resource-limited setting.

Participants believed antibiotics to be overused and misused in both Syria and Jordan, mainly due to the availability of over-the-counter antibiotics, extensive and prolonged antibiotic consumption without indication, and prematurely discontinuing antibiotic treatment. These factors were seen as the main contributors to ABR development, which is in line with WHO’s views on the global development of ABR [13]. We sensed that these factors were considered as out of the participants’ hands.

The participants’ perceptions of the interrelationship between HAI and ABR were not as clear as could maybe have been expected. No direct links between HAI and the development and spread of ABR were described by any of the participants. It could be that the participants are so used to the levels of both ABR and HAI that they do not mention it further.

The main challenges linked to ABR brought forward by the participants were difficulties managing isolation cases and high costs of the antibiotics needed for treatment. These factors are both acknowledged as challenges in the fight against ABR [13].

The study results should be interpreted with some caution. English is neither the first language of the researchers nor the participants. However, as English is the language used in the project, we consider the risk of misinterpretations as relatively low. Furthermore, a qualitative study method with a small sample size from one specific hospital limits the generalizability. However, the results may be transferred to similar resource-limited settings, primarily in the same geographical region.

## 5. Conclusions

We conclude that in a setting with high numbers of patients with severe injuries and limited resources, physicians do not see HAI as something they can prioritize. The knowledge gained by this study could provide insights for the allocation of resources and development of wound care and hygiene protocols in limited-resource settings. The participants’ split views on the significance of HAI and ABR development suggest that there is more to explore on these complex topics. Interventional studies could help assess the impact of training packages to improve hand hygiene compliance. Focus group discussions with members of the medical team could be used to evaluate preventive measures taken, and study how and to what extent HAI and ABR affect the day-to-day care.

## Figures and Tables

**Table 1:** Demographic characteristics of the ten participating physicians.

Characteristic	*n*/Years
Sex, *n*	
Male	6
Female	4
Age, years	
Mean	37
Range	27–63
Origin, *n*	
Jordan	6
Non-Jordan	4
Medical specialty, *n*	
General or orthopedic surgeon	5
Not specialized	5
Working experience, years	
Mean	11
Range	2–35

**Table 2:** Overview of the theme, categories, and sub-categories that emerged from the content analysis.

Theme	Category	Sub-Category
Interrelationship between behavior, healthcare-associated infections, and the development of antibiotic resistance	Healthcare-associated infections	Behaviors and conditions influencing the spread of infection
		Lack of consensus on the problem
	Antibiotic resistance	Causes of antibiotic resistance
		Challenges related to antibiotic resistance

## Appendix A. Introductory Questions and Probing Areas

1. How did you manage your last patient with a war-associated soft tissue wound? Was this management similar or different compared to a typical case?
  *Probe:* Debridement, dressing type, dressing change frequency.
2. What indication for antibiotic prescription was there for your last patient with a war-associated soft tissue wound? Was this management similar or different compared to a typical case?
  *Probe:* Common pathogens, differentiating between contaminating and infecting organisms, signs of infection, cultures, use of antibiotic prescription guidelines.
3. How would you treat infection in war-associated soft tissue wounds?
  *Probe:* Antibiotic types, the use of broad-spectrum antibiotics.
4. What is your view on antibiotic resistance?
  *Probe:* Possible causes, impact of antibiotic use, the use of broad-spectrum antibiotics.
5. Have you ever prescribed antibiotics when there is no real indication, just in case?
  *Probe:* Influence by the patient or relatives.
6. How do people obtain antibiotics?
  *Probe:* Without a prescription, Jordan, Syria, types of antibiotics, broad-spectrum antibiotics.
7. What is your view on antibiotic resistance at Ar Ramtha hospital?
  *Probe:* Interviewees will be presented with antibiotic resistance data from Ar Ramtha hospital, possible causes, impact of antibiotic use, the use of broad-spectrum antibiotics.
8. What is your view on hand hygiene?
  *Probe:* Gloves, disinfectants, in-between patients, dressings.
9. What is your view on healthcare-associated infections?
  *Probe:* Possible causes, role of hand hygiene.
10. How do you view the cooperation with the laboratory, regarding wound cultures?
  *Probe:* Sending cultures, patient information on referrals, receiving culture results, laboratory involved in treatment decisions, desired cooperation with the lab.
11. Is there something you would like to add?
